# Lactic acid bacteria derived extracellular vesicles: emerging bioactive nanoparticles in modulating host health

**DOI:** 10.1080/19490976.2024.2427311

**Published:** 2024-11-13

**Authors:** Mohan Li, Bingyong Mao, Xin Tang, Qiuxiang Zhang, Jianxin Zhao, Wei Chen, Shumao Cui

**Affiliations:** a State Key Laboratory of Food Science and Resources, Jiangnan University, Wuxi, Jiangsu, China; b School of Food Science and Technology, Jiangnan University, Wuxi, Jiangsu, China; c International Joint Research Laboratory for Maternal-Infant Microbiota and Health, Jiangnan University, Wuxi, Jiangsu, China; d National Engineering Research Center for Functional Food, Jiangnan University, Wuxi, Jiangsu, China

**Keywords:** Lactic acid bacteria derived EV (LAB-EVs), biogenesis, isolation and purification methods, fFunction, diseases

## Abstract

Lactic acid bacteria derived extracellular vesicles (LAB-EVs) are nano-sized and carry a variety of biological cargoes. LAB-EVs have proven to be potential mediators of intercellular communication, serving not only the parental bacteria but also the host cell in both physiology and pathology. LAB-EVs are therapeutically beneficial in various diseases through a cell-free strategy. Particularly, EVs secreted from probiotics can exert health-promoting effects on humans. Additionally, the excitement around LAB-EVs has extended to their use as nano-sized drug carriers, since they can traverse biological barriers. Nevertheless, significant challenges in terms of isolation, characterization, and safety must be addressed to ensure the clinical application of LAB-EVs. Therefore, this review emphasizes the isolation and purification methods of LAB-EVs. We also introduce the biogenesis, cargo sorting, and functions of LAB-EVs. The biological regulatory factors of LAB-EVs are summarized and discussed. Special attention is given to the interaction between LAB-EVs and the host, their ability to maintain intestinal homeostasis, and the immunity and inflammation they induce in diverse diseases. Furthermore, we summarize the characterization of LAB-EV cargoes by advanced analytical methods such as proteomics. Finally, we discuss the challenges and opportunities of LAB-EVs as a means of diagnosis and treatment in clinical translation. In conclusion, this review scrutinizes current knowledge and provides guidelines for proposing new perspectives for future research in the field of LAB-EVs.

## 1. Introduction

Lactic acid bacteria (LAB) constitute a diverse taxonomic assemblage proficient in fermenting carbohydrates to produce lactic acid.^1^ Primarily classified within the phyla Firmicutes, Actinobacteria, and Bacteroidetes, this group encompasses a broad spectrum of genera, totaling approximately 45. LAB exhibit a ubiquitous presence across various ecological niches, including traditional fermented foods, human and animal microbiomes, and environmental reservoirs such as soil and water.^1,2^ Key genera within this bacterial cohort include *Lactobacillus*, *Lactococcus*, *Leuconostoc*, *Streptococcus*, et al. Within the human gastrointestinal tract, Firmicutes and Bacteroidetes represent the predominant phyla, with *Lactobacillus*, *Streptococcus*, and *Bifidobacterium* emerging as principal genera of LAB. As symbiotic entities, LAB significantly influence regulatory dynamics and intervention strategies pertinent to health maintenance and the onset and progression of diseases in biological hosts, including plants, animals, and humans.^3–5^ LAB elicit beneficial effects on hosts through diverse mechanisms, although the precise pathways through which these effects manifest remain elusive. In the early twenty-first century, researchers begun to focus on the metabolites and intercellular communication mechanisms of LAB. Within this context, the emergence of LAB-derived extracellular vesicles (LAB-EVs) represents a burgeoning area of investigation.

Extracellular vesicles (EVs) constitute a class of membranous nanostructures, typically ranging in diameter from approximately 20 to 800 nanometers. Considerable evidence suggests that various bacteria have the capacity to release EVs as part of their normal metabolic functioning.^6^ Extensive research focused on bacterial EVs has unveiled their proficiency in encapsulating a myriad of biological payloads, including DNA, RNA, proteins, lipids, and other metabolites, thus endowing them with pivotal roles in maintaining human health. Notably, LAB-EVs serve as pivotal mediators in cell-cell communication networks and have emerged as a promising therapeutic modality for addressing human ailments and diseases. Furthermore, their ability to traverse the bloodstream and breach numerous biological barriers enables them to influence distant tissues or remain localized within the vicinity of secretion, thereby facilitating autocrine or paracrine modulation.^7^

The discovery of LAB-EVs will greatly impel the revelation of the substance basis of LABs’ benefits to the human body. LAB-EVs boast numerous advantageous attributes including cell-free characteristics, minimal toxicity, exceptional biocompatibility, low immunogenicity, non-replicative nature, and precise cellular targeting capabilities.^8,9^ Notably, LAB-EVs exhibit the remarkable ability to concentrate active substances to a high degree. Leveraging their membranous structure, LAB-EVs provide a protective environment for internal active constituents, ensuring their stability during transportation and preserving their bioactivity at an elevated level. Multiple lines of inquiry underscore the multifaceted biological functionalities inherent to LAB-EVs. These vesicles actively participate in a spectrum of biological processes within their parental bacteria, encompassing but not confined to maintenance of ecological niches through facilitation of cooperation, competition, or antagonism with other microbial species.^10^ Moreover, LAB-EVs exert regulatory control over the activities of recipient cells by conveying intercellular signals and bioactive payloads.^11–13^

## 2. The biogenesis, isolation methods, and composition of LAB-EVs

### 2.1. LAB-EV biogenesis

EVs are one of many offspring among bacteria’s vital movements and are heterogeneous due to their formation largely depending on the parent cells/bacteria and growth conditions.^14^ EVs can be secreted throughout the entire growth cycle of bacteria.^15,16^

Biogenesis of EVs from mammalian cells and Gram-negative bacteria has been extensively studied over the past few decades. Mammalian cell-derived EV biogenesis is complex and generally involves pathways dependent on the endosomal sorting complex required for transport (ESCRT) and ESCRT-independent pathways. The ESCRT mechanism is a multi-molecular system composed of various proteins that interact with ubiquitinated cargo to promote the formation of intraluminal vesicles (ILVs).^17^ The ESCRT-independent pathway is coordinated by the neutral sphingomyelinase family, enzymes that convert sphingomyelin in lipid rafts into ceramide.^17^ The resulting ceramide interacts to form a large micro-area, inducing ILV germination and multivesicular body (MVB) formation. MVB transport within cells toward the plasma membrane relies on interactions with actin and cytoskeletal microtubules and is regulated by numerous proteins, notably the GTPase family of Rab proteins. Additionally, the presence of lipid rafts, particularly cholesterol, is crucial during microvesicle (MV) formation, which occurs through outward budding from the mammalian cell membrane. Notably, the activity of acidic sphingomyelinase and the conversion of sphingolipids to ceramides are also involved in MV production, a process similar to that of neutral sphingomyelinase in extracellular biogenesis. Ceramide, a cone-shaped lipid, can induce membrane bending and trigger MV release.^18,19^

Research on Gram-negative bacteria suggests that outer membrane vesicles (OMVs) may result from weakened interactions between the outer membrane and peptidoglycan during cell wall renewal, leading to OMV germination and release in a relaxed state.^20^ Another theory posits that peptidoglycans and misfolded proteins increase periplasmic space, thereby facilitating EV release.^21^

Extracellular vesicles were initially thought to be exclusive to Gram-negative bacteria due to the thick peptidoglycan (PG) cell wall of Gram-positive bacteria. However, early research in the 1990s confirmed that Gram-positive bacteria can also produce EVs.^22^ This discovery has garnered significant attention, particularly regarding LAB-EVs, due to their health-promoting effects from probiotics.^23–26^ Despite this interest, research on Gram-positive bacterial EVs remains limited. This review focuses on introducing the biogenesis mechanisms of EVs secreted by Gram-positive bacteria, especially those from *Lactobacillus*. Proteomic analysis of EVs from Gram-positive bacteria using mass spectrometry methods has revealed the presence of penicillin-binding proteins and autolysin, suggesting a relationship between cell wall modification and vesicle release.^27^

**Figure 1.**
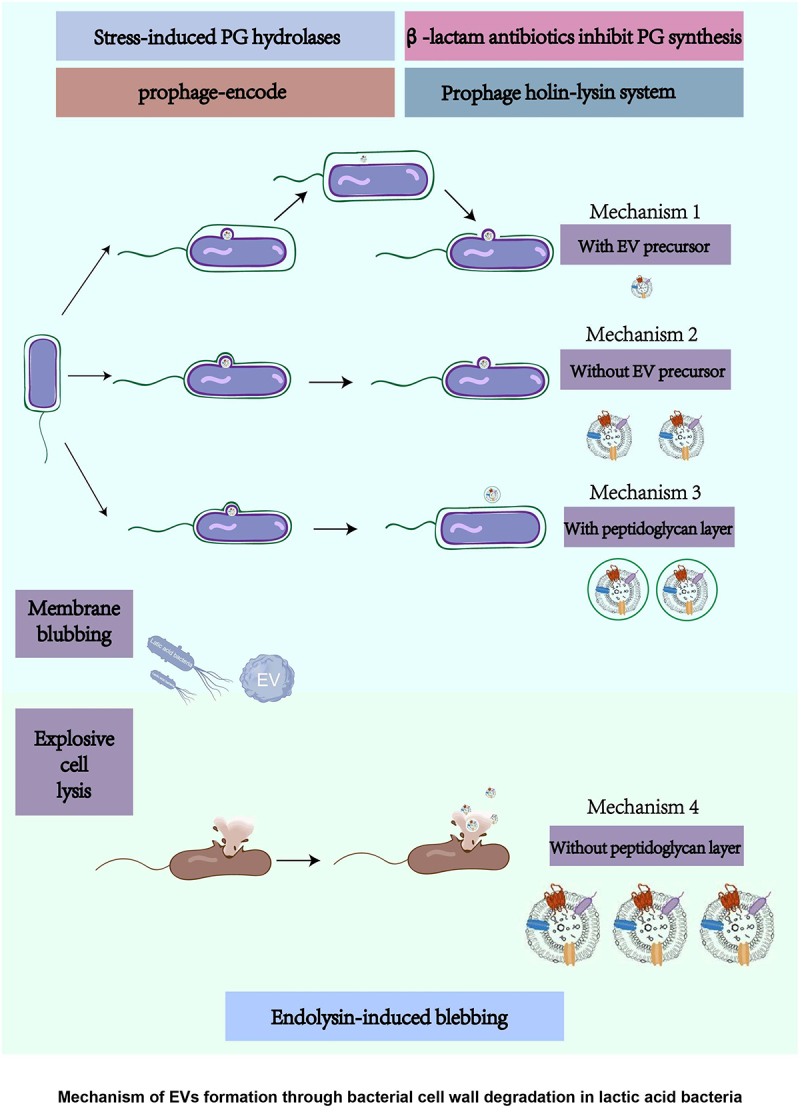
Biogenesis mechanism of EVs from Gram-positive bacteria.

Current evidence supports the hypothesis that bacterial cell wall-degrading enzymes and prophage activation weaken the peptidoglycan (PG) layer and promote EV release. The biogenesis mechanisms of EVs from LAB are illustrated in Figure 1. Mechanisms include *β*-lactam antibiotics inhibiting PG synthesis, prophage-encoded and stress-induced PG hydrolases digesting the bacterial wall, and the prophage holin-lysin system facilitating EV production.^28^ Choline insertion into the cell plasma membrane allows PG hydrolase (endolysin) to enter and digest the bacterial wall.^28–30^ The biogenesis of LAB-EVs comprises four mechanisms: membrane blebbing with or without EV precursors and explosive cell lysis. When EV precursors form, they exist between the cytoplasmic membrane and the PG layer, potentially resulting in smaller EVs due to space constraints within the inner wall zone (IWZ) (Figure 1 Mechanism 1). EVs without precursors can be secreted through PG layer pores after plasma membrane foaming (Figure 1 Mechanism 2). Additionally, some researchers have observed EV structures coated by the PG layer using super-resolution microscopy (Figure 1 Mechanism 3).^31^ The release of autolysin and antibiotics can relax the PG layer and promote vesicle release. Endolysin-triggered explosive cell lysis is typically induced by genotoxic stress (Figure 1. Mechanism 4). During bacterial growth, Cao and colleagues demonstrated that adding appropriate amounts of isopropyl-β-D-thiogalactopyranoside (IPTG) and kanamycin to the culture can successfully promote the production and release of AST-carrying outer membrane vesicles (AST-OMVs).^32^ In addition to the disruption of cell wall components, the disruption of cell membrane components may also facilitate the secretion of EVs by Gram-positive bacteria. Wang et al. reported that alpha-type phenol-soluble modulins (PSMs) promote EV biogenesis in *Staphylococcus aureus* by disrupting the cytoplasmic membrane.^33^ Ronan K. Carroll and his colleague also found that peptidyl-prolyl cis-trans isomerase (PPIase), which regulates the production of alpha phenol-soluble modulin (αPSM) peptides, can increase EV secretion Extracellular vesicle biogenesis and functions in gram-positive bacteria,^27,34^ suggesting that αPSM is a key factor in temperature-dependent EV secretion mechanisms. There is concern that disrupting the integrity of the cell wall and membrane, although it can increase vesicle release, may impact the viability of the bacteria themselves. Therefore, when utilizing EV secretion inhibitors, researchers should verify whether these inhibitors impact the parent bacteria.

The generation of vesicles occurs not only under normal physiological conditions but also when cells/bacteria are stimulated to secrete vesicles containing specific bioactive substances. For example, when attacked by pathogens (bacterial pathogens and fungi/oomycetes), plant cells can release various types of EVs for defense.^35^ Silicate ions can also induce the production of highly bioactive EVs in endothelial progenitor cells.^36^ BEVs contribute to microbial survival or competition. BEVs containing iron-binding factors from *Mycobacterium tuberculosis*,^15,37^ *Streptomyces azureus* M110,^38^ and *Staphylococcus aureus* contribute to bacterial survival under iron-limited conditions. Additionally, bacteria produce large amounts of lactic acid during their metabolic processes, creating an acidic environment that can stress and damage the bacteria, leading to vesicle release as a protective mechanism.

It is noteworthy that experiments have shown that heat-inactivated bacteria lack the capability to secrete EVs, indicating that vesiculation relies on metabolically active living cells.^39–41^ This suggests that vesicle biogenesis in Gram-positive bacteria is not coincidental but a vital mechanism. Recent studies using super-resolution microscopy have revealed mechanisms of extracellular vesicle biogenesis in Gram-positive bacteria. Three principal mechanisms have been proposed: membrane blebbing and explosive cell lysis. The importance of cell wall degradation in the biogenesis of Gram-positive bacterial EVs was emphasized, with pores in the peptidoglycan layer assisting in EV formation.^31^

### 2.2. Preparation, characterization, and preservation methods of LAB-EVs

#### 2.2.1. Isolation and purification methods

Despite the high interest in LAB-EVs research, many aspects remain unexplored, necessitating the development of advanced detection and characterization methods to dissect LAB-EVs structure, biological function, and material basis. There are multiple challenges in actual EV production, including safety inspection, large-scale production, efficiency, etc. LAB-EVs are heterogeneous in size and composition and may be contaminated with unwanted materials and cellular debris from the culture medium. To precisely explore LAB-EVs functional characteristics, obtaining high-purity LAB-EVs is essential. Consequently, isolation and purification processes for LAB-EVs are indispensable and critical. Numerous techniques for EV isolation and purification have been developed, including ultracentrifugation (UC), density gradient centrifugation (DGC), ultrafiltration (UF), size-exclusion chromatography (SEC), tangential flow filtration (TFF), and others.

Among these methods, UC is a routine method for vesicle extraction, as it can reduce operational procedures, decrease experimental costs, and shorten time. Briefly, LAB-EVs are subjected to a series of centrifugations with increasing speeds (more than two rounds). Next, filters remove cell debris and contaminants, and the supernatant is continuously centrifuged, culminating in a focus on the precipitate (pellet). For example, *L. plantarum* Q7-EVs are first centrifuged at 8000 g for 30 min, and after filtration through a 0.22 μm filter, the supernatant is concentrated using an ultrafiltration tube and ultracentrifuged at 100,000 g for 2 h.^42^

UF has also been used as a conventional EV isolation method and acts as an excellent approach. Briefly, the core factor of UF is a membrane filter, which serves as a mini sieve to allow smaller particles to flow through while capturing particles that exceed a certain size threshold. For instance, to obtain MVs, the supernatant is first collected, and then a 100 kDa hollow-fiber membrane is used to concentrate EVs from the supernatant.^43^ Hong and colleagues collected and concentrated EVs using a 100 kDa Vivaflow 200 followed by high-speed centrifugation.^44^ It appears that filtration and concentration are essential and indispensable procedures to acquire purer EVs.

DGC is a density-based purification method. Sucrose and iodixanol are the most commonly used DGC media for purifying EVs. Compared to sucrose, iodixanol has characteristics such as being isotonic, inert, and having low viscosity. Therefore, the required centrifugation time is shorter, and the osmotic pressure remains constant, so the volume and density of EVs do not change during the centrifugation process. DGC can effectively remove impurities from samples, making it very suitable for downstream experiments that require high EV purity. However, this method’s operation is complex and the yield is low. EVs are usually isolated from ultrafiltration liquid by bottom-up Optiprep DGC.^45^

**Figure 2.**
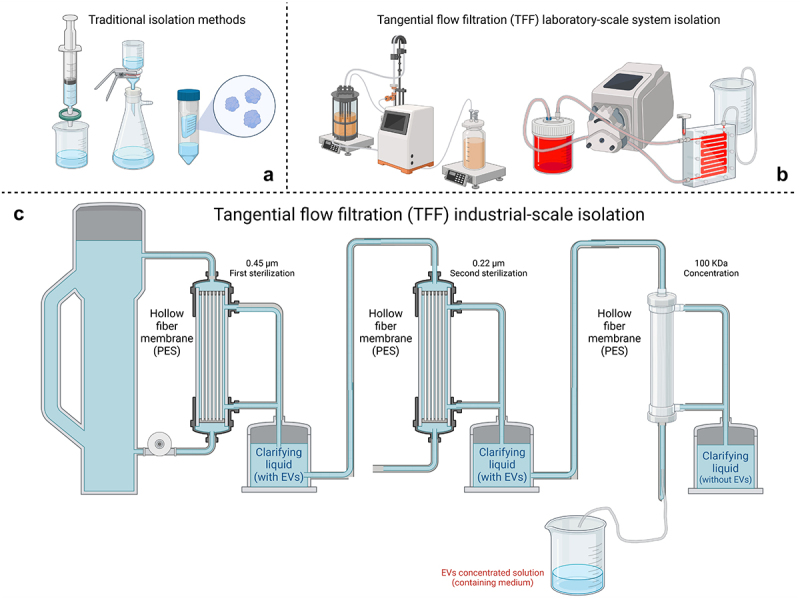
IFF isolation technology and traditional EV separation technology, including traditional isolation methods (a), tangential flow filtration (TFF) laboratory-scale system isolation (b), and tangential flow filtration (TFF) industrial-scale isolation (c) (created with BioRender.com).

TFF technology has been applied in the delipidation of human serum particles since the 1970s.^46^ However, it was not until 2020 that TFF was widely applied for EV isolation. TFF has two modes of membrane filtration devices: membrane package and hollow fiber. Membrane package filters are usually used for monoclonal antibody concentration because they can provide higher flux. Hollow fiber is very useful when the target entity is sensitive to shear force. In the separation process of EVs, it is generally recommended to maintain shear forces below 2000/s. Using a hollow fiber membrane filtration device enables effective control of shear forces, as the pump pressure can be maintained as low as 0.5 bar. Consequently, TFF employing hollow fiber membrane filtration devices has garnered significant trust and adoption for EV isolation due to its ability to mitigate shear forces while ensuring efficient separation. Ultracentrifugation and TFF exhibit comparable abilities to enrich consistent populations of EVs, characterized by similar size distributions encompassing particles up to 200 nm. Nonetheless, TFF surpasses ultracentrifugation by yielding significantly higher quantities of EVs, making it better suited for large-scale research endeavors.^47–49^ The TFF approach^11^ (seen in Figure 2b,c) is suitable for preparing and applying large volumes of LAB-EVs, which can purify and collect LAB-EVs, perform robust downstream ultrafiltration and microfiltration, accelerate multiple testing/process processing, ensure accurate process definition and operational consistency, and thus achieve faster development of EV extraction processes. Accurate and repeatable control and complete parameter recording can be achieved. Moreover, compared with traditional isolation methods (Figure 2a), the TFF method holds particular significance in making *in vivo* experiments involving EVs more practicable and achievable.

With technological advancements, increasing methods are being used to address the low activity and yield of EVs, enabling them to exhibit higher activity and significant biological functions. For instance, high-performance anion exchange chromatography purification of probiotic bacterial extracellular vesicles enhances purity and anti-inflammatory efficacy.^50^ Collectively, these approaches exploit differential physical properties via density, size, solubility, as well as membrane outer protein, to isolate and purify these EVs from parental bacteria and other non-EV components. However, it should be noted that these approaches are not perfect and still pose several technical challenges. For this reason, we list the methods commonly used in the laboratory to extract and purify EVs (Table S1). When using a 0.22-μm/0.45-μm filter to separate EVs, which are often described as EVs, it is important to be cautious. Such filters may intercept most EVs, leaving primarily exosomes. The choice of membrane material for the filter is also critical. Commonly used filter membranes include EPS and PVDF materials due to their low protein adsorption characteristics, which help minimize losses of EVs.^51,52^

#### 2.2.2. Characterization methods

Accurate characterization of EV particle size, quantity, and surface morphology is vital for their subsequent *in vitro* and *in vivo* applications. Classic methods include dynamic light scattering (DLS), nanoparticle tracking analysis (NTA), atomic force microscopy (AFM),^53^ transmission electron microscopy (TEM), cryo-TEM, scanning electron microscopy (SEM), and flow cytometry (FC) using a TDI CCD-camera in high gain mode. NTA, in particular, can reveal the density and particle size distribution of EVs. For example, Mina et al. observed that *Streptococcus pneumoniae* secretes varying concentrations of EVs at different growth stages, with yields of 10^11^ particles/mL during the stationary phase at 24 hours and up to 10^12^ particles/mL during the death phase at 48 hours.^54^ Additionally, single-particle detection techniques are evolving and have been successfully applied in EV research.^55,56^

Fluorescent dye labeling of EVs is a necessary characterization method for tracking EVs *in vivo* and *in vitro* .^57–59^ There are two common fluorescent labeling methods: membrane protein fluorescent probe labeling (e.g., CFSE for green fluorescence, mCherry for red fluorescence, GFP for green fluorescence) and lipophilic fluorescent dye labeling (most commonly used). The membrane protein fluorescent probe has a large volume, high steric hindrance, and low abundance and strong heterogeneity of extracellular vesicle membrane proteins, greatly limiting the efficiency of fluorescence labeling. The latter (lipophilic fluorescent dye labeling) is advantageous due to its simplicity, high fluorescence intensity, and small molecular size, making it suitable for efficient labeling and downstream detection of EVs *in vitro* and *in vivo*. Popular lipophilic fluorescent dyes include DiI (orange fluorescence), DiO (green fluorescence), DiD (red fluorescence), and DiR^52^ (deep red fluorescence). However, lipophilic fluorescent dyes can aggregate at higher concentrations, forming nanoparticles similar in size to EVs and potentially causing false positive signals. This can lead to overestimation of EV size and quantity. Therefore, controls lacking fluorescent dye-labeled vesicles are crucial when studying biological distribution to discern whether observed signals in cells result from vesicle-mediated cargo transport or from free dyes, aggregated dyes, or non-EV particle delivery. Recent studies suggest using LPS and outer membrane protein OmpA as biomarkers for characterizing EVs derived from Gram-negative bacteria. For Gram-positive bacteria, lipoteichoic acids (LTAs) can serve as biomarkers for EVs.^60^

### 2.3. Composition of LAB-EVs

There is a broad consensus that LAB-EVs are involved in intercellular signaling transfer and act as a communication medium between bacteria and their environment. EVs contain a diverse array of bioactive molecules such as proteins, lipids, and nucleic acids, which can independently exert biological effects or modulate biological processes in recipient cells.^61^ Bacterial EVs play a regulatory role in shaping microbial community structure and function and serve as a vehicle for sharing active molecules between bacteria for competition and survival. The cargoes within EVs are crucial for immune evasion and modulation in the host. *Lactobacillus*-derived EVs exhibit various biological activities by altering the microenvironment. For example, after inducing *Lactobacillus acidophilus* with lactacin B-inducing peptides, the secreted EVs, enriched in putative bacteriocins encoded by the *lab* operon, can inhibit pathogen colonization.^53^ The secretion of EVs by LAB underscores their adaptive responses to environmental challenges and their active role in microbial communities. Numerous studies have revealed that EVs are replete with microbe-associated molecular patterns (MAMPs),^62^ proteins, nucleic acids, toxins, signaling molecules, enzymes, and antibiotic resistance factors. The cargo enables EVs to identify the appropriate docking and fusion sites. Beyond their roles in pathogenesis, bacterial EVs are implicated in multiple functions, including nutrient acquisition, quorum sensing, and biofilm formation ^63(p1)^. EVs also play a wide spectrum of biological roles in survival,^64^ nucleic acid transfer, and antibiotic resistance. The unique membrane structure of these vesicles and their contained cargoes directly influence their properties.

#### 2.3.1. Proteins

Proteins are universally acknowledged as the primary bioactive substances that confer functionality to EVs. Comparative analyses of the compositional content of proteins within EVs have consistently demonstrated a higher protein content relative to other substances.^60^ The protein composition of EVs is not identical to that of their parent bacterial strain, resulting in distinct biological functionalities.^53^ Additionally, the content and categorization of proteins within EVs secreted at different growth stages show significant variation.^16^ Proteomic analyses using LC/MS-MS have revealed proteins in EVs involved in various biological processes, including membrane formation, host cell interaction, and immune regulation. Probiotic-derived EV proteins play crucial roles in promoting colonization of their parent bacterial strains in the human gastrointestinal tract by facilitating adhesion, modulating immunity, and enhancing bacterial survival within the host niche.^65^ The protein composition of *Lactobacillus* enterovirus proteins was analyzed by liquid chromatography and tandem mass spectrometry, including *Lactiplantibacillus plantarum*, *Limosilactobacillus fermentum*, and *Lactobacillus gasseri*. The predominant EV proteins are associated with biological processes such as catalytic activity, gluconeogenesis, cell wall biosynthesis, and glycolysis. Motif enrichment analysis indicated that proteins derived from *Lactiplantibacillus plantarum* and *Lactobacillus fermentans* were rich in serine motifs.^66^ Furthermore, proteins such as glyceraldehyde-3-phosphate dehydrogenase, citrate lyase alpha chain, and enolase were the most abundant in EVs derived from *L. plantarum*, *L. fermentum*, and *L. gasseri*, respectively. A study on *Staphylococcus aureus* revealed that compared to the parent bacterial proteome, EV proteins are generally more positively charged, contain more small residues, and have fewer aromatic, aliphatic, and hydrophobic amino acids.^67^ BEVs released from biofilms sequester antibiotics, facilitating bacterial survival and biofilm development. Biofilm matrix proteins present in EVs confer viscoelastic properties to cells along with adhesion-related proteins.^68^ This review presents methods for characterizing proteins in LAB-EVs and specific protein molecules identified in table form (see Table 1).

**Table 1.** Protein characterization techniques for LAB-EVs.

Origin	Protein Characterization Techniques	Constituent	Refs
*Lactobacillus lactis*	SDS-PAGE & peptide mass fingerprinting	Pyruvate kinase, arginine deiminase, and ornithine transcarbamylase	^69^
*Streptococcus equi*	Proteomes LC-MS/MS (orbitrap VELOS) & immunoprecipitation	IgG, amino acid permease, amidase, putative exported protein, anti-phagocytic factor H binding protein(se18.9), peptide ABC transporter substrate-binding protein et al.	^70^
*Streptococcus pyogenes*	Proteomes LC-MS	Pyogenes, NAD-glycohydrolase	^71^
*Lacticaseibacillus paracasei*	Western Blot	P40 and P75	^72^
*Bifidobacterium longum* subsp. *longum*	Proteomes LC-MS	Phosphoketolase, GroEL, elongation factor Tu (EF-Tu), phosphoglycerate kinase, transaldolase (Tal), and heat shock protein 20 (Hsp20)	^73^
*Escherichia coli* O83	Proteomes LC-MS/MS (label-free)	Flagellar proteins FlgL 1 and 3, FlgE, FlgG, FliC and LPS	^74^
*Bifidobacterium longum* subsp. *longum* AO44	Liquid chromatography-tandem mass spectrometry	ABC transporters, transmembrane permease proteins, nucleotide-binding proteins, and highly specific periplasmic solute-binding proteins	^75^
*Lactobacillus johnsonii*	Proteomes LC-MS/MS (4D label-free) & western blot	Glutamine synthetase	^76^
*Escherichia coli* (BL21)	Proteomes LC-MS/MS	Cell outer-membrane protein, Periplasm protein, Cytoplasm protein, Cell inner-membrane protein	^77^
*Bacillus coagulans*	Proteomes LC-MS/MS	Flagellin, LTA	^60^
*Escherichia coli* DH5α		LPS, OmpA, flagellin	
*Lactiplantibacillus plantarum* BCRC 10,069	Proteomes LC-MS/MS	Glyceraldehyde-3-phosphate dehydrogenase, Glucose-6-phosphate isomerase, L-lactate dehydrogenase et al.	^66^
*Limosilactobacillus fermentum* SWP-AFFS02		Citrate lyase alpha chain, Ornithine carbamoyltransferase, DNA starvation/stationary phase protection protein et al.	
*Lactobacillus gasseri* BCRC 17,615		Enolase, Glucose-6-phosphate isomerase, Endopeptidase, ABC transporter, ATP-binding protein et al.	
*Limosilactobacillus reuteri* DSM 17,938	Proteomes lipid-based protein immobilization (LPI) methodology	LPxTG anchored 5′-nucleotidase, Phosphoketolase, 6-Phosphogluconate dehydrogenase, Aminopeptidase PepN et al.	^78^
*Limosilactobacillus reuteri* BG-R46		LPxTG anchored 5′-nucleotidase, Glyceraldehyde-3-phosphate dehydrogenase, Dextran sucrase, Peptidoglycan hydrolase，ATP synthase subunit alpha et al.	
*Lactobacillus lactis* ssp. *cremoris* FM-YL11	Proteomes LC-MS/MS	Phage head protein, scaffolding protein, Phage tail proteins et al.	^28^
*Lactococcus lactis*	Peptide mass fingerprinting	Pyruvate kinase, arginine deiminase, ornithine transcarbamylase et al.	^69^
*Lactiplantibacillus plantarum*	Proteomes LC-MS/MS	Lysozyme, cell wall amidase lytH, aryl-sulfate sulfotransferase, nucleoside 2-deoxyribosyltransferase et al.	^79^
*Lactococcus lactis*	Western Blot	Caveolin‑1β	^80^
*Streptococcus pyogenes* strains SSI-1	Proteomes LC-MS/MS	Streptolysin O, NAD-glycohydrolase, C5a peptidase, exotoxin type A/C, and streptokinase, D-alanyl-lipoteichoic acid biosynthesis protein, LPXTG cell wall anchor domain-containing protein et al.	^71^
*Lactobacillus johnsonii* N6.2	Proteomes LC-MS/MS and Western Blot	Sdp_SH3b2, Sdp_SH3b6, Enolase et al.	^81^
*Lactobacillus gasseri* BC12	Proteomes LC-MS/MS	ATP synthase subunit beta, Phosphonates import ATP-binding protein PhnC, ATP synthase subunit b, Enolase 2, Elongation factor Tu et al.	^82^
*Lactobacillus crispatus* BC5		ATP synthase subunit beta, Phosphonates import ATP-binding protein PhnC, ATP synthase subunit b, 50S ribosomal protein L4 et al.	
*Limosilactobacillus reuteri* BBC3	Proteomes LC-MS/MS	Glucosyltransferase GtfG, Serine protease, Elongation factor Tu, Inositol polyphosphate phosphatase 1 et al.	^83^
*Lactiplantibacillus plantarum* WCFS1	Proteomes LC-MS/MS	DNA-binding protein, L-lactate dehydrogenase 1, Oligopeptide ABC transporter, substrate binding protein et al.	^8^
*Lactiplantibacillus plantarum* BGAN8	Proteomes MALDI-TOF/MS and LC-MS/MS	Amino acid/peptide ABC transporters er al.	^84^

#### 2.3.2. Nucleic acid

EVs are considered an important vehicle for gene transfer across species and for horizontal gene transfer. For example, EVs can carry DNA and various types of RNA molecules including mRNA, tRNA, rRNA, and non-coding RNAs (ncRNAs), predominantly microRNAs (miRNAs), long non-coding RNAs (lncRNAs), and circular RNAs (circRNAs). Numerous studies have shown that ncRNAs are intimately involved in human health and the onset and progression of various diseases.^85–87^ Yu et al. identified small RNAs by sequencing sRNAs from *Lactiplantibacillus plantarum*-derived extracellular vesicles and assessed vesicular sRNA expression levels using quantitative reverse transcription-polymerase chain reaction (RT-PCR). Subsequent transfection experiments with synthetic sRNA71 mimetics demonstrated significant downregulation of Tp53 expression in HEK293T cells by binding to the 3‘UTR of Tp53 mRNA.^88^

Although evidence suggests that bacterial-derived EVs carry nucleic acids, the biological functions of these nucleic acids within EVs still require further elucidation and validation.

#### 2.3.3. Lipids

The EVs of LAB consist of phospholipid bilayers. This distinctive membrane structure allows the encapsulated cargo to maintain high activity levels and be transported over long distances to terminal tissues or organs. Studies have reported that the lipid composition of EVs varies depending on their source, which affects their preferential absorption by specific bacteria.^89^

Lipidomic analyses have revealed that the lipid classes enriched in EVs include diacylglycerol (DG), triacylglycerol (TG), phosphatidylcholine (PC), phosphatidylserine (PS), and lysophosphatidylserine (LPS), all of which are present at levels more than two-fold higher in EVs than in parent bacteria.^35^ Additionally, EVs contain a higher proportion of glycerophosphoethanolamines (PE), glycerophosphoglycerols (PG), cardiolipins, and monogalactosyldiacylglycerol (MGDG) compared to their original bacteria.^81^

#### 2.3.4. Others

Among LAB, lipoteichoic acids (LTA) are embedded in their cell wall during the logarithmic phase. However, a unique case exists in *Lactobacillus gasseri JCM* 1131, where LTA is exposed on the membrane vesicles.^90^ Metabolites and effector molecules regulate the function of target cells. Bacterial EVs can serve as carriers for the secretion of hydrophobic quorum sensing molecules, mediate communication within bacterial communities, and control important processes such as disease or biofilm formation. Notably, Gram-negative bacterial EVs exhibit low endotoxicity and low immunogenicity due to the absence of acylated lipopolysaccharide^91^ (LPS).

### 2.4. Sorting and uptake in LAB-EVs cargoes

#### 2.4.1. Sorting in LAB-EVs

Although the mechanisms behind cargo sorting into mammalian cell-derived EVs remain unclear, several possibilities have been proposed. For example, protein molecules may be sorted into EVs in a ubiquitin-dependent manner with the aid of the endosomal sorting complex required for transport (ESCRT).^9^ Additionally, tetralipoproteins on the membrane may facilitate protein distribution in EVs. However, the secretion mechanism of bacterial EVs differs from that of mammalian cell-derived EVs. The selective sorting mechanism during the formation of bacterial EVs is driven by electrostatic interactions and may involve specific proteins that recognize curvature, as seen with αPSMs in *Staphylococcus aureus*, which assist in EV release from the plasma membrane. The protein composition of BEVs derived from *Staphylococcus aureus* is predominantly positively charged compared to the whole cell proteome, containing more small residues and fewer aromatic and aliphatic groups. In Gram-positive bacteria, CMV protein cargo has an overall positive charge, suggesting that charge characteristics play a significant role in cargo selection.^92^ This implies that physical and chemical properties are crucial in EV cargo sorting from Gram-positive bacteria.^67^

Proteomic studies comparing protein composition in the outer membrane (OM) and OMVs have shown significant differences between mucin-culture medium (MCM) and polysaccharide-culture medium (PCM). However, OM protein composition from MCM does not significantly differ from OMVs from PCM, indicating that *Bacteroides multiforme* actively sorts proteins into OMVs. In *Bacteroides multiforme*, lipoproteins carrying the LES motif are actively loaded into OMVs.^59^ Current research on the secretion mechanisms of LAB or Gram-positive bacterial EVs is limited, but the sorting mechanisms of Gram-negative bacterial EVs may provide insights for speculation or prediction.

In summary, there are numerous challenges and limitations in studying the cargo classification mechanism in LAB-EVs. Due to the limitations of existing research, we can only make reasonable speculations based on the existing sorting mechanism of EVs derived from mammalian cells. For example, RNA binding proteins are involved in regulating the sorting/secretion of miRNAs in some EVs. Li et al. found that RNA binding protein YBX1 is a key protein required for selectively sorting non-coding RNA fragments hY4F that are significantly enriched in EVs.^93,94^ Thus, identifying the types of proteins and understanding their functions can greatly assist in sorting cargo in future LAB-EVs.

#### 2.4.2. The uptake of LAB-EVs by mammalian cells

The endocytosis and exocytosis processes of EVs are reversible and rapid, involving repeated cycles of movement between different cells. EVs serve as a secretion mechanism, allowing bacterial active compounds to be transported over long distances within protected environments.^10^ Cargo transfer to recipient cells involves multiple mechanisms, including direct membrane fusion, receptor-ligand interaction, endocytosis, micropinocytosis, clathrin-mediated processes, lipid raft-mediated pathways, and phagocytosis.^95^ As previously mentioned, fluorescently labeled EVs combined with various uptake pathway inhibitors are commonly used to characterize uptake experiments through reverse validation. It remains unclear whether targeting of cells, tissues, and organs is solely related to signaling molecules on bacterial EVs or also involves selective uptake by receptor cells. Current research on non-bacterial sources of EVs indicates that different types of polysaccharides, lipids, proteins, etc., impact the uptake of EVs by recipient cells to varying degrees and through different mechanisms.^96^ However, it is confirmed that if membrane proteins (especially transmembrane proteins) on EVs are inhibited or reduced, the uptake efficiency of EVs by recipient cells is significantly decreased.^97^ Numerous *in vitro* and *in vivo* experiments have confirmed the ability of mammalian cells to uptake LAB-EVs, yet the precise mechanisms remain elusive.^98,99^ The uptake of extracellular vesicles derived from LAB by receptor cells may be influenced by various factors, which warrant further in-depth study:

1. Existence and expression level: Specific receptors on the surface of receptor cells may bind to molecules on extracellular vesicles derived from LAB. The presence and expression level of these receptors directly affect uptake efficiency.
2. Surface molecules of EVs: The molecular characteristics of the vesicle surface determine interactions with recipient cells.^62,100^ This may involve specific proteins, sugars, or other biomolecules.
3. Environmental conditions: Environmental conditions such as temperature and pH value influence the efficiency of cell uptake of extracellular vesicles. Optimal conditions promote interactions between receptors and vesicles.
4. Size and shape: The size and shape of extracellular vesicles may affect their interaction with recipient cells. Certain sizes or shapes may be preferentially uptaken by some cells.
5. Membrane characteristics: Properties of the cell membrane, including fluidity and permeability, can also affect the uptake process.
6. Intracellular signaling pathways: Signaling pathways within receptor cells may regulate the response to external vesicles.
7. Physiological state: The physiological state of cells, such as nutritional status and cell cycle phase, may also influence the uptake process.

Considering these factors can enhance our understanding of the mechanisms and regulation involved in the uptake of EVs derived from LAB by receptor cells. This research is crucial for comprehending microorganism-host cell interactions, disease treatment, and other applications.

## 3. LAB-EVs on host health

**Figure 3.**
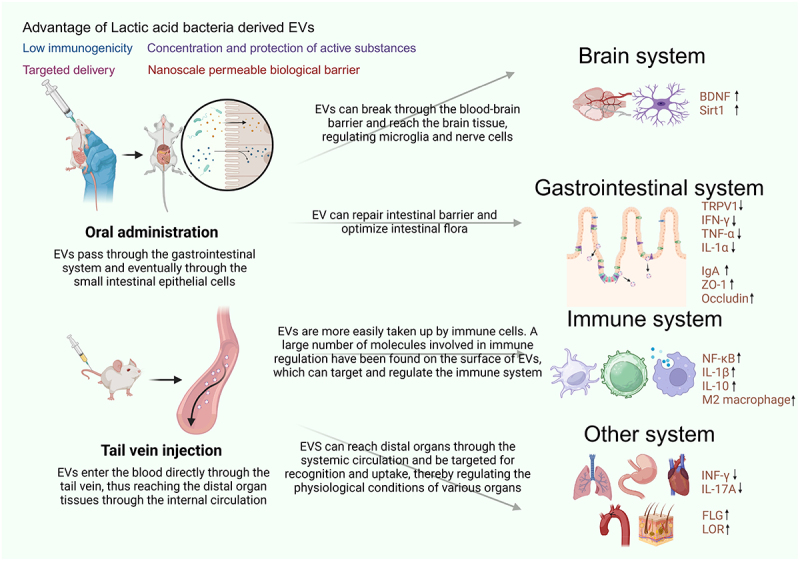
Advantages and functions of lactic acid bacteria-derived EVs (created with BioRender.com).

LAB-EVs have various health benefits, such as neutralizing bacteriophage infections, preventing the spread of virulence factors,^101,102^ regulating immune response and inflammation. Growing insights into EVs’ roles in physiology and pathology reveal their potential in diagnostics and therapy, highlighting their significant clinical promise. EVs are low immunogenicity since they do not contain nucleus and are non-replicable (highlighted in Figure 3). The impact of bacterial EVs on health and disease is gradually being revealed. LAB-EVs have been reported to play a vital role in preventing and treating several diseases, including colitis, cardiovascular diseases, neurological diseases, diabetes, bone loss, and skin diseases. Table 2 summarizes the functions of LAB-EVs, functional molecules within EVs, and associated diseases. Notably, the functionality of EVs is determined by the cargo they transport. A large part of their components come from the original cells, represent the state of those cells and plays an essential role in exerting EVs’ functions. In addition, to ensure the safety of LAB-EVs in regulating human health, we have summarized some necessary preparations and related research directions (seen in Table S2).

**Table 2.** Mechanisms of LAB-EV regulation on the host.

Parental bacterium	*In Vitro* experiment	*In Vivo* experiment	Disease	Biological functions of EVs	Activity cargoes (effective substances)	Biodistribution	Molecular mechanism	Refs
*Lactiplantibacillus plantarum*	HaCaT cells were treated with 0.1, 1 and 10 μg/mL EVs for 12 hours to evaluate the immunogenicity of Lp EVs	Lp EV was administered orally 12 hours before each Sa EV administration	Atopic Dermatitis	*In vitro*: Decrease IL-6 from keratinocytes and macrophages *In vivo*: Reduce IL-4	Sorted proteins	\	\	^103^
kefir grain *Lactobacillus*	Caco_2_ was stimulated with 2 μg/mL of TNF-α; 1 × 10^9^ particles/mL of kefir-derived *Lactobacillus* EV were cultured with Caco-2 cells for 24 h	Rectal administration of 0.1 mL of 2.5% TNBS to the anus for model, EVs were then orally administrated for 10 d	Inflammatory bowel disease	In vitro: reduced the expression of IL-8 and TNF -α in TNF treated cells In vivo: Kefir-derived Lactobacillus EV mixture reduced inflammatory response and decrease the serum MPO	\	EV reached the colon	NF-κB↓	^104^
*Lactiplantibacillus plantarum*	HEK 293 T cells (2 × 106 cells) (Sigma-Aldrich) were co-transfected with miR-101a-3p mimic and luciferase reporter plasmids	tMCAO mice injected with LEVs through the tail vein for 3 consecutive days	Acute ischemic stroke	\	101a-3p	\	101a-3p/c-Fos/TGF-β axis	^105^
*Lacticaseibacillus paracasei*	A549 cells were treated with 1 mg/mL LPS with or without LpEV in 2 doses (1 and 10 ug/mL) for 24 h	Mice were orally administered with 30 or 300 mg/kg LpEV using a gastric gavage needle	Eosinophilic asthma	Oral administration of LpEV reduced airway resistance and inflammation in mice, AHR as well as neutrophil counts and CXCL1 and IL-17 production in the BALF were significantly decreased	Threonine-tRNA ligase and enolase， D-(-)-tagatose, palmitoleic acid, and dodecanoic acid	Lungs and even brain within 24 h	JNK/IL-8/Neutrophils	^99^
*Bifidobacterium longum subsp*. *longum* AO44	T cell were then supplemented with 0.05 mm β-Mercaptoethanol and antiCD3 (0.5 μg/ml) for suboptimal activation of the T cells. 100 μl of cells were plated in each well of a 96-well plate and were added with 100 μl of diluted fractions (1:250 dilution) or vesicles (1:50-1:31,250 dilution)	EVs were introduced to SPF mouse splenocytes	Immune-modulatory, anti-inflammatory	Ex vivo: Stimulate the secretion of IL-10	ABC transporters, transmembrane permease proteins, nucleotide-binding proteins, and highly specific periplasmic solute-binding proteins	Spleen	\	^75^
*Lactobacillus johnsonii*	100 μL of cells (1 × 10^5^ cells/mL) in serum-free medium were seeded in the upper chamber of 5-micron Transwells while LJ-MVs (5 μg/μL) were introduced in the lower chamber	*L. johnsonii* (5 × 10^9^ CFU per mouse) was administered by intraperitoneal injection every day and LJ-MVs (9.5 × 10^9^ particles per mouse) were administered tail intravenously every two days for a period of 42 days	Osteoarthritis (OA)	EVs mitigate inflammation, cartilage damage, and pain associated with OA, while simultaneously promoting the M2/M1 ratio in synovial macrophages. LJ-MVs treatment reduced secretory concentrations and the expression levels of MCP-1 in M1-like macrophage	Glutamine synthetase (GS)	\	Macrophage glutamine synthetase/mTORC1	^76^
*Limosilactobacillus mucosae*	\	Mice fed with *L. mucosae*-derived EVs	Diarrheal	EVs decreased the level of IL-1β, IL-6, IL-8, TNF-α, LPS in the serum, modulated macrophage phenotypes to counteract diarrheal disease symptoms	DNA, RNA, and proteins	\	Akt/NF-κB	^106^
*Lacticaseibacillus paracasei*	Aβ42 plus Lpc-EV (10 μg per ml, final) were treated starting 24 h after HT22 cells transfection	Lpc-EV was orally administered to mice at a dose of 2.27 mg/Kg/day by drinking water from 6.5 months of age until sacrifice at 8.0 months of age	Alzheimer’s disease	*In vitro*: EV restored BDNF, Nt3, Nt4/5, TrkB receptor, and increased the level of Mecp2 and Sirt1; *In vivo*: EV alleviated Aβ accumulation and neuroinflammatory responses in the brain, and mitigated cognitive decline in Tg-APP/PS1 mice	\	\	MeCP2/Sirt1/MMP	^12^
Maternal fecal	\	The EVs were injected into the tail vein of the mice	\	EV can break through the placental barrier	Protein and DNA	Muscle, fetus, lung, heart, liver and brain	\	^107^
*Lacticaseibacillus paracasei* PC-H1	After 16 h of incubation, the cells were administrated with different LpEVs concentrations of 100 μg/mL, 150 μg/mL, and 200 μg/mL for 48 h.	200 μl PBS (containing mixture of 1.5 × 10^6^ HCT116 cells and 200 μg/mL LpEVs) was injected subcutaneously into each nude mouse	Colorectal cancer	*In vitro*: LpEVs inhibited the proliferation, migration, invasion and promote apoptosis of colorectal cancer cells; *In vivo*: LpEVs inhibited the growth of CRC xenograft in nude mice and promoted tumor apoptosis	\	\	PDK1/AKT/Bcl-2	^108^
*Lactococcus lactis*	Dendritic cells were treated with 10 μg/mL EVs for 24 h	Allergic asthmatic mice were intranasally treated with 10 μg EVs	Allergic asthma	*In vivo*: *L. lactis*-EV treatment shifted immune responses from Th2 to Th1 by stimulating dendritic cells to produce IL-12	Pyruvate kinase as well as arginine deiminase and ornithine transcarbamylase	\	\	^69^
*Limosilactobacillus reuteri* BBC3	HD11 cells (5 × 10^5^ cells/mL), were pretreated with LrEVs (10 μg/mL) for 12 h	The birds were given by gavage the purified LrEVs (200 μg/bird) in 200 μL protectant (5% skim milk)	Inflammatory bowel disease	*In vivo*: LrEVs suppressed the LPS-induced expression of pro-inflammatory genes (TNF-α, IL-1β, IL-6, IL-17 and IL-8), and improved the expression of anti-inflammatory genes (IL-10 and TGF-β) in the jejunum; *In vitro*: LrEVs reduced the gene expression of TNF-α, IL-1β and IL-6 by suppressing the NF-κB activity, and enhanced the gene expression of IL-10 and TGF-β in LPS-activated chicken macrophages	Glucosyltransferase, serine protease, 60 kDa chaperonin, elongation factor Tu and inositol polyphosphate phosphatase 1	\	NF-κB↓	^83^
*Lactiplantibacillus plantarum*	THP1 cells were treated with 10 μg/mL LEVs for 48 h	\	\	EV induced anti-inflammatory M2 macrophage polarization	\	\	\	^11^
*Lacticaseibacillus paracasei*	HT29 cells were serum starved for 3 h and incubated with LpEVs for the indicated times; RAW 264.7 cells were treated with LpEVs (0.1, 1, 10 μg/mL) for 12 h.	A total of 5 mg of *L. paracasei* EVs suspended in phosphate-buffered saline were administered to mice from day 0 to day 12 by oral gavage	Colitis	*In vivo*: LpEVs reduced LPS-induced inflammation in HT29 cells and decreased the activation of inflammation-associated proteins, such as COX-2, iNOS and NFκB, as well as nitric oxide; *In vitro*: LpEVs protected against DSS-induced colitis by reducing weight loss, maintaining colon length, and decreasing the disease activity index	\	Stomach, large intestine, lung, brain and small intestine	ER stress-NF-κB↓	^98^
*Lactobacillus gasseri* BC12	MT-4 cells were treated with *L. gasseri* BC12 derived EVs (5 × 10^8^ particles per mL)	\	HIV-1 infection	EVs reduce HIV-1 entry/attachment to target cells for suppressing HIV-1 infection	Envelope proteins and elongation factor Tu	\	\	^82^
*Lacticaseibacillus rhamnosus* GG	MC38 cells were handled with 100 ug ml^−1^ of LGG-EV for 36 h	Mice were administered EV by gavage (5 mg kg − 1)	Colorectal cancer	*In vitro*: LGG-EV inhibited the proliferation of colorectal cancer cells and promotes apoptosis *In vivo*: LGG-EV improves the composition of the intestinal microbiota	\	Intestine, stomach, small intestine, colon	\	^109^
*Lactobacillus murinus*	RAW 264.7 cells were treated with 20 μg/mL LmEVs for 12 h	Mice were oral gavaged with 50 μg LmEVs per day.	Intestinal toxicity	*In vitro*: LmEVs enhanced barrier function of DONchallenged intestinal epithelial cells; *In vivo*: LmEVs conferred intestinal barrier repair via activation	\	\	TLR2	^110^
*Lactobacillus johnsonii*	J774A.1 cells were coincubated with LjEVs (20 μg/mL) for 12 h	Mice were orally administered 50 μg of LjEVs daily	Diarrheic	*In vitro*: L. John-derived EVs (LjEVs) enhanced M2 macrophage polarization for the repair of intestinal barrier *In vivo*: LjEVs alleviated ETEC K88 treatment caused intestinal inflammatory injury	\	\	MAPK\NLRP3\ASC	^111^
*Lacticaseibacillus paracasei*	Cells grown to 70 ~ 80% confluence were treated with Lpc-EV (10 μg/ml) for 24 h	Lpc-EV was administered to mice via the intraperitoneal route, each dose being 6 μg of Lpc-EV in 100 μl of injection volume	Emotional behavior	*In vitro*: Lpc-EV counteracted GC-induced decreased expression of BDNF, Nt3, Ngf, and TrkB; *In vivo*: Lpc-EV relieved stress-induced depressive-like behavior	\	Brain, liver and kidney	MKP-1\Fkbp5\MECP2	^112^

### 3.1. LAB-EVs and brain health

EVs’ significance for human health is a burgeoning field in biomedical research and EVs can cross biological barriers like the blood-brain barrier (BBB), access brain tissue and benefit the brain health. It is reported that EVs secreted by *Lactiplantibacillus plantarum* (*L. plantarum*) can cross the blood-brain barrier and be internalized by neurons after intraperitoneal injection.^105^ A recent study also showed that *L. plantarum*- derived EVs enhanced the expression of brain-derived neurotrophic factor (BDNF) and Sirt1 in HT22 cells, and ameliorated stress-induced depressive-like behaviors in CRST-treated mice.^113^ In addition, *Lacticaseibacillus paracasei* (*L. paracasei*)-derived EVs can cross the blood-brain barrier and affect neuronal cells, thereby modulating amyloid-induced changes in the mouse brain.^112^ EVs from kimchi-derived LAB could block the inflammatory response in LPS-stimulated microglia by inhibiting the extracellular signal-regulated kinase (ERK) and p38 signaling pathways.^114^ However, not all bacteria-derived EVs would benefit the brain health. *Streptococcus equi* subsp. *zooepidemicus*-derived EVs cross the BBB and disrupting the murine BBB by inducing autophagic endothelial cell death.^115^ The various health effects may be dependent on the interior components of EVs from different LAB. In addition, researchers found that EVs from *Paenalcaligenes* (a genus isolated from elderly individuals and aging mice) can penetrate the brain through blood and vagus nerves, and vagotomy inhibited *Paenalcaligenes*-EVs infiltration into the hippocampus,^116,117^ indicating that EVs can reach the interior of brain through the vagus nerves and blood.

### 3.2. LAB-EVs and immune regulation

Numerous studies have shown that EVs derived from LAB can be taken up by host M cells and enter the intestinal lamina propria, thereby regulating their downstream reactions.^118,119^ Kim et al. found that EVs derived from *L. plantarum* APsulloc 331,261 can induce the differentiation of human monocyte THP-1 cells into an anti-inflammatory M2 phenotype, particularly M2b, by inducing biased expression of cell surface markers and cytokines associated with M2 macrophages.^11^ *L. plantarum*-derived EVs can also activate the innate immune system of intestinal epithelial cells (IECs) and upregulate the expression of host defense genes in IECs to protect the host from pathogen invasion.^8^ In addition, LAB derived EVs could regulate the production of pro-inflammatory cytokines (TNF-α, IL-1β, and IL-6) as well as anti-inflammatory cytokines (IL-10, IFN-γ, and IL-12) by lipopolysaccharide (LPS) stimulated macrophages (RAW264.7) in different ways.^79,114^ Moreover, EVs derived from *Lactococcus* shifted the immune response from a Th2 to a Th1 bias by inducing dendritic cells to produce IL-12 and serum-specific IgG4 levels in asthma patients were significantly lower than those in the healthy control group.^69^ EVs derived from *Pediococcus pentosaceus* fostered the polarization of bone marrow-derived macrophages toward an M2-like phenotype, which was dependent on Toll-like receptor 2 (TLR2) signaling.^120^ As shown in the above reports, LAB derived EVs have exerted the immune regulatory effects. However, few studies have revealed the key substances in EVs. *Lacticaseibacillus rhamnosus* (*L. rhamnosus*) derived EVs were reported to regulate immunity,^121–123^ some of which contain lipoteichoic acid (LTA) and biologically active proteins (p40 and p75). Notably, EVs derived from *L. rhamnosus* JB-1 contain lipoteichoic acid (LTA), which can activate TLR2 and induce the expression of immune regulatory interleukin-10 in dendritic cells in an internalization-dependent manner.^123^

LAB are not a strict taxonomic definition, but a common term referring to a group of microorganisms that can produce lactic acid. Besides the generally regarded LAB (such as *Lactobacillus*, *Lactococcus*, *Pediococcus*, *Leuconostoc* and *Streptococcus*), some researchers also consider *Bifidobacterium* as LAB in a broad sense,^124–129^ which could also produce lactic acid besides acetic acid through heterologous lactic acid fermentation.^130^ EVs derived from certain *Bifidobacterium* were also reported to be able to regulate the immunity^75,131–133^ and the effects were strain specific. *Bifidobacterium longum* subsp. *longum* (*B. longum*) strain AO44 derived EVs exerted outstanding anti-inflammatory effects, which could induce IL-10 secretion from both splenocytes and dendritic cell (DC)-CD4+ T cell co-cultures. Proteomic analysis has revealed that these EVs are rich in ABC transporters, quorum sensing proteins, and extracellular solute-binding proteins, which have shown more significant anti-inflammatory effects compared with other *B. longum* strains.^75^ However, Morishita et al. found that EVs from *B. longum* increased the production of proinflammatory cytokines in TLR2-expressing mouse macrophage-like RAW264.7 cells and mouse dendritic DC2.4 cells.^132^ The different immunity-regulatory effects may be related to the interior components of EVs.

### 3.3. LAB-EVs and intestinal health

When it comes to improving intestinal health, there have been many reports on probiotics alleviating intestinal inflammation, repairing intestinal barriers, and regulating intestinal immunity.^134–137^ However, in many cases, the material basis and mechanism of probiotics in improving intestinal health have not been clearly elucidated. In recent years, researchers have also been focusing on LAB derived EVs and found that they can promote intestinal health.

*In vitro* experiments demonstrated that adding *L. paracasei*-derived EVs to LPS-stimulated HT29 cells significantly reduced levels of inflammatory cytokines such as IL-1α, IL-1β, IL-2, and TNF-α, and decreased inflammation-related proteins (cyclooxygenase-2, inducible nitric oxide synthase, and nuclear factor kappa B) as well as nitric oxide production.^98^ Kefir grain *Lactobacillus*-derived EVs could regulate the inflammatory response by reducing the production of inflammatory cytokines in Caco-2 cells induced by tumor necrosis factor-alpha (TNF-α), indicating of their potential as a treatment for inflammatory bowel disease.^104^ *In vivo* studies showed that oral administration of *L. paracasei*-derived EVs prevented DSS-induced colitis by reducing weight loss, preserving colon length, and decreasing the disease activity index.^98^ EVs derived from kefir grain *Lactobacillus* ameliorate intestinal inflammation by modulating pro-inflammatory pathways (NF-κB pathway) and enhancing intestinal barrier function.^138^ In addition, LAB derived EVs could also regulate the intestinal immunity. EVs from *Latilactobacillus sakei* subsp. sakei NBRC15893 stimulated the production of immunoglobulin A (IgA) from murine intestinal Peyer’s patch cells via TLR2 signaling activation.^119,139^ EVs from *Lactobacillus murinus* activated TLR2 to promote polarization of M2 macrophages and release IL-10, thereby alleviating deoxynivalenol-induced intestinal barrier damage.^110^ Some LAB derived EVs also showed potential inhibitory effect on colon cancer growth.^140,141^ These EVs are internalized by colon cancer cells and significantly inhibit their proliferation, migration, and invasion in a concentration-dependent manner. After 48 h of co-incubation with colon cancer cells, *L. paracasei* derived EVs significantly induced apoptosis, as indicated by Annexin V/PI double staining. Further research confirms that the pro-apoptotic role of EVs is mediated through the PDK1/AKT/Bcl-2 signaling pathway.^108^

*Limosilactobacillus reuteri* (*L. reuteri*) is an important source species of EVs, which has been proved to play an important and positive role in regulating intestinal health.^83,142,143^ *L. reuteri* DSM 17,938 derived EVs could reduce the intestinal leakage caused by enterotoxigenic *Escherichia coli* in a dose-dependent manner, upregulate the pro-inflammatory factor IL-1β in peripheral blood mononuclear cells (PBMCs) and IL-6 but also suppress the secretion of IFN-γ and TNF-α in PBMCs challenged by *Staphylococcus aureus* .^143^ Moreover, the EVs also had an antagonistic effect on the pain receptor transient receptor potential vanilloid 1 (TRPV1) in a model with primary dorsal root ganglion cells from rats. Further studies on the investigations of material basis for EVs functions revealed that vesicular proteins and nucleic acids are essential for the immunoregulation of EVs from *L. reuteri* BBC3^e^, while lipids and proteins are important structural components of EVs derived from *L. reuteri* DMS 17,938.^142^

### 3.4. LAB-EVs and skin health

Oral administration of EVs may be absorbed into the bloodstream and transported to the distal end to exert their effects. Several studies found that LAB derived EVs may be beneficial for skin health. *L. plantarum*-derived EVs treatment decreased interleukin (IL)-6 secretion from keratinocytes and restored cell viability. In *S. aureus*-induced atopic dermatitis (AD) mice, *L. plantarum*-derived EVs administration reduced epidermal thickening and IL-4 levels.^103^ Additionally, Jo et al. found that *L. plantarum* derived EVs modulated the mRNA expression of extracellular matrix (ECM)-related genes, while suppressing wrinkle formation and pigmentation in clinical trials.^144^ In addition, EVs derived from *Lactobacillus druckerii* inhibited collagen I/III and α-SMA expression in fibroblasts and reduced hypertrophic scar formation in a scleroderma mouse model while also promoting skin cell proliferation, new blood vessel formation, and wound healing.^145^ *B. bifidum*-derived EVs can also trigger the expression of filaggrin (FLG) and loricrin (LOR) in human primary epithelial keratinocytes,^146^ which has a protective effect on the skin barrier. Besides, a recent study demonstrated that EVs from *Leuconostoc horzafimine* stimulated hair growth in dermal papilla cells of human hair follicles when applied to the human scalp.^147^ These EVs were found to reduce cell apoptosis and enhance hair growth through the Wnt/β-catenin signaling pathway.^147^ These studies indicated that certain LAB derived EVs are beneficial for skin and hair growth. Unfortunately, the effector factors from the EVs haven’t been revealed.

### 3.5. LAB-EVs and host health in other aspects

Besides the above-mentioned health effects, LAB-EVs have also been reported to be beneficial for host health in other aspects. Bacterial EVs from healthy pregnant women’s amniotic fluid resemble those in the maternal gut, indicating they can cross the placental barrier and affect fetal development.^107^ This may be related to the fact that EVs are known to cross mucosal^123^ and blood-brain barrier,^148^ though the mechanisms are not fully understood. EVs from *Lactobacillus crispatus* (*L. crispatus*), commonly found in the vagina, improved Akt phosphorylation and mitigated oxidative stress-induced aging and death in placental cells.^149^ A recent study showed that EVs from *L. crispatus* BC3 and *Lactobacillus gasseri* (*L. gasseri*) BC12 could protect tissues and cells from HIV-1 infection, which is mediated by reducing virus attachment/entry to target cells and is associated with specific proteins and metabolites present in the EVs.^82^ Additionally, certain LAB-EVs were also reported to be associated with liver, spinal cord and lung health. *L. rhamnosus* derived EVs provoke cytotoxic effects on HepG2 cells *in vitro*, increase the apoptotic index (*bax/bcl-2* expression ratio) and activate the apoptosis and cancer cell death.^150^ EVs from *L. reuteri* had an antagonistic effect on vanilloid receptor 1, a transient receptor potential of pain receptors, in a rat primary dorsal root ganglion cell model.^143^ Oral administration of *L. paracasei*-derived EVs reduced airway resistance and inflammation. Subsequent studies revealed that *L. paracasei* and its three metabolites (dodecanoic acid, palmitoleic acid, and D-(-)-tagatose) significantly inhibited JNK phosphorylation/IL-8 production *in vitro* .^99^

Although the research on LAB-EVs is currently in a flourishing stage, there are still challenges in basic research and clinical translation, such as EVs separation technology, specificity for host health regulation, and optimization of EVs storage and transportation. Compared with EVs secreted by tissue cells, research on LAB-EVs is still in its early stages, and future clinical studies with strict design are needed to confirm their diagnostic and therapeutic value. We believe that in the near future, the disease prevention and control achievements of LAB-EVs can benefit more people in sub-healthy and unhealthy.

## 4. Conclusions and future perspectives

**Figure 4.**
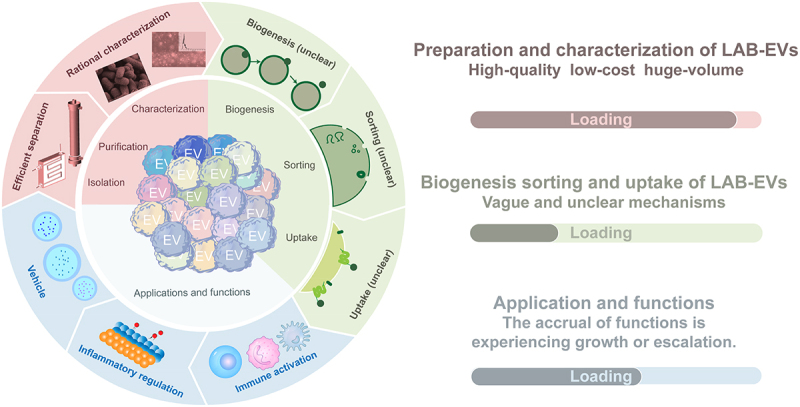
The entire process of LAB-EVs from isolation to application.

LAB research has advanced significantly, particularly in elucidating the potential probiotic attributes of LAB. Although numerous studies have confirmed the probiotic functions of LAB, a gap remains in understanding the material basis of these functions. The investigation of LAB-EVs has gained traction over the past decade, offering insights into their functional roles. Evidence suggests that LAB’s functionality is likely mediated through its secreted EVs. LAB-EVs can concentrate nucleic acids, proteins, and other bioactive molecules. Their protective membrane structure enables them to deliver bioactive effectors to distant organs and tissues, including the brain, skin, intestine, and other vital tissues, thereby exerting functional impact. Despite burgeoning research endeavors in the field of EVs, significant gaps persist in our understanding of biogenesis, cargo sorting, host uptake, and the material foundation underlying their biological actions (as shown in Figure 4).

While the advantages of EVs are well-established, practical challenges persist in their application across diverse domains. In exploring LAB-EVs mechanisms, a multifaceted guidance framework is necessary. One significant aspect is achieving scalable and cost-effective methods for EV production. It is crucial to increase the yield and purity of LAB-EVs while ensuring the bioactivity and safety of their cargo. Technologies aimed at boosting LAB-EVs production must prioritize maintaining the integrity and efficacy of the active payloads within these EVs, safeguarding against harmful cargo that could adversely impact human health. Balancing yield enhancement with cargo quality preservation is imperative for advancing LAB-EVs’ practical applicability in various industries and therapeutic settings. Additionally, characterizing LAB-EVs remains challenging; variations in methodologies can affect EV purity and result reliability, especially when exploring EVs *in vivo*. If fluorescent dyes like Dil, DiO, or Cys are used in LAB-EVs preincubation processes, it is essential to ensure that free fluorescent dyes are completely removed from LAB-EVs. In clinical practice, challenges related to characterizing and standardizing EV-based therapies persist due to the heterogeneity of EV populations and variability in cargo composition and biological activity.

Of note, many studies have employed technical means to identify EVs as effective substances, typically through the use of vesicle secretion inhibitors. These inhibitors include calpain peptide, Y27632, pantothiamine, imipramine, GW4869, Manumycin A, dynasore, U0126, clopidogrel, imatinib, NSC23766, dimethylamiloride, glibenclamide, indomethacin, chlorpromazine, cytochalasin D, and trimethoprim. However, some studies have not considered the impact of these inhibitors on cell viability – an important factor that warrants in-depth exploration. In *in vivo* models, significant differences arise in the delivery of EVs to the body through various routes, such as oral or intranasal administration, or intravenous and intraperitoneal injections. Different methods for administering bacterial EVs include gavage,^98,151^ intravenous injection, subcutaneous injection, and intraperitoneal injection.^77^ To date, there have been relatively few targeted research and characterization on the final destination and residence site of LAB-EVs among different administration methods.^133^ Additionally, natural EVs exhibit low absorption by receptor cells, leading to off-target effects and rapid clearance from circulation, which significantly reduces the utilization rate of EVs. A key to therapeutic application is enhancing the uptake rate of EVs through engineering technologies. Limited attention has been directed toward studying the production of EVs by LAB *in vivo*. A recent publication by Ou et al.,^152^ in the *Journal of Extracellular Vesicles*, sheds light on the ability of bacteria to generate EVs within the intestinal environment. Nonetheless, a considerable gap remains in understanding the specific mechanisms underlying the functional role of these EVs within the intestine. Regulating EVs in situ *in vivo* poses a significant challenge compared to the relative ease of modulating EVs *in vitro*. For example, manipulation of EV properties can be achieved by adjusting culture times and conditions. Therefore, exploring strategies to regulate LAB-EVs in situ, ensuring the production of beneficial EV populations for the host, is a critical direction for future research. Notably, the specific uptake mechanism of LAB-EVs remains unclear; further research should focus more on host cell membranes and transmembrane proteins to address uptake mechanisms.

Moreover, challenges related to the storage and stability of EVs must be addressed. EVs are inherently fragile and prone to degradation, thus, optimized storage conditions and delivery strategies are necessary to preserve their integrity and bioactivity. Although EVs hold vast potential for various applications, overcoming these practical challenges is essential for realizing their full translational potential and facilitating their widespread adoption in research, industry, and clinical practice. With this realization, LAB-EVs have the potential to be developed as a new type of postbiotic in functional foods or health products due to their key biological functions.

EVs play multifaceted roles in therapeutics, acting as pharmaceutical agents and vehicles for drug delivery.^153^ They also serve as targets for drug action or as biomarkers in various applications. EVs possess unique structural features with hydrophilic and hydrophobic microdomains that allow them to encapsulate both water-soluble and water-insoluble drugs.^154^ Their bilayer membrane structure provides excellent biocompatibility with biological matrices, facilitating integration and interaction within complex biological environments.

Delivering large and lipid-insoluble drugs remains a significant challenge in drug delivery. There are mainly two different effective drug loading strategies in EVs, including exogenous loading (i.e. after EV isolation) and endogenous loading (i.e. during EV biogenesis).^155^ Engineering LAB-EVs, an endogenous loading strategy, offers a new avenue for developing therapeutic agents and vaccines. Of note, exogenous loading is easier than endogenous loading. The scientific community has developed a spectrum of approaches to facilitate the exogenous loading of cargo into EVs, each with its own advantages and limitations. Electroporation applies an electric field to transiently permeabilize the EV membrane, thereby enabling the introduction of cargo molecules.^156^ This method has the potentials to achieve high transfection efficiency, and the risk of altering the integrity of the EVs. Simple incubation represents a more passive approach, where cargoes are allowed to interact with the EVs under controlled conditions, with the expectation that they will be incorporated into the EVs over time.^157^ This technique is often appreciated for its simplicity and minimal perturbation to the EV structure. Sonication employs ultrasonic waves to disrupt the EV membrane, creating transient pores through which cargo can be loaded.^158^ Extrusion and freeze-thawing are physical methods involving mechanical disruption of the EV membrane.^159,160^ Extrusion typically involves forcing the EVs through a membrane with a defined pore size, while freeze-thawing cycles exploit the physical stress induced by freezing and thawing to create openings in the membrane. The efficacy of these techniques varies, and the choice often depends on the specific cargo, the nature of the EV, and the desired outcome of the loading process. Each technique presents a unique set of challenges and opportunities for optimization, and a comprehensive understanding of their mechanisms is crucial for the advancement of EV-based therapeutic and diagnostic applications.

Modified and engineered EVs exhibit enhanced efficiency, specificity, and safety for diverse disease therapies. For example, the toxicity of drug-loaded mixed EVs to cancer cells is increased, and pH-sensitive drugs are released under acidic conditions, facilitating targeted drug delivery to acidic cancer environments.^161^ Wu et al. successfully designed an innovative hepatic-targeted vesicle system encapsulating with fucoxanthin (Glycyrrhetinic acid-modified *Lacticaseibacillus paracasei* EVs loading fucoxanthin, GA-LpEVs-FX).^162^ *In vivo*, the administration of GA-LpEVs-FX has demonstrated a significant downregulation of key proteins associated with lipogenesis, including fatty acid synthase, acetyl-CoA carboxylase, and sterol regulatory element-binding protein 1. This downregulation has been correlated with a substantial amelioration of lipid metabolism disorders.^162^

Strategies also include co-formulating drugs with specific proteins that activate transporter proteins to facilitate drug entry into target cells or tissues. Another approach involves encapsulating well-characterized drugs into EVs to traverse biological barriers more efficiently. These strategies exploit the unique properties of EVs to enhance drug bioavailability and therapeutic efficacy. Hence, MVs provide an ideal platform for delivering bacterial cell surface proteins to host cells.^115^

There is still a considerable distance to traverse in bridging the gap between fundamental research and clinical application in the field of LAB-EVs. Moreover, current research lacks comparative studies on the properties of EVs secreted by different LAB strains during their growth stages. Bearing this in mind, future research should focus more on the timing of EV extraction and the foundational aspects of cargoes. Additionally, the regulatory landscape for EV-based therapies is in a state of flux, necessitating the establishment of clear guidelines and frameworks to ensure their safety, efficacy, and quality.

## Supplementary Material

4 Supplementary file.docx

## Data Availability

Research data are available upon request.
