# Supplementary material for: Lactic acid bacteria derived extracellular vesicles: emerging bioactive nanoparticles in modulating host health
Source: Gut Microbes. 2024 Nov 13;16(1):2427311. doi: 10.1080/19490976.2024.2427311 (PMC11572086; doi:10.1080/19490976.2024.2427311)
Supplement: 4 Supplementary file.docx [file KGMI_A_2427311_SM3278.docx]

**Supplementary file**

**Lactic acid bacteria derived extracellular vesicles: Emerging bioactive nanoparticles in modulating host health**

Table S1. Isolation and purification methods for EVs.

Table S2. Necessary preparations and related research directions to ensure the safety of LBA-EVs.

###### Table S1. Isolation and purification methods for EVs.

| Method | | Intro | | Advantage | | Disadvantage | | Refs |
| --- | --- | --- | --- | --- | --- | --- | --- | --- |
| Ultracentrifugation (UC) | | Remove strains and their fragments by low-speed centrifugation, and precipitate EV by high-speed centrifugation | | No need to mark EV, convenient operation, suitable for extraction of a large number of samples | | The process is time-consuming and high-speed and repeated centrifugation will destroy the EV structure | | (Fan et al., 2024; Cheng et al., 2021) |
| Density gradient centrifugation (DGC) | | Combining ultracentrifugation with sucrose density gradient centrifugation | | EV can be separated from non-EV components, resulting in higher concentration of EV | | The process is time-consuming and cumbersome | | (Harrison et al., 2021) |
| Ultrafiltration (UF) | | Filter the solvent and small molecules to intercept EV through the membrane gap | | Simple operation, low cost, enrichment and high efficiency, no impact on EVs biological activity | | EVs are considerable loss; Deformation of EV membrane; Block the filter hole; Reduce the service life of the membrane; Decrease efficiency | | (Rodovalho et al., 2021; Tzipilevich et al., 2017) |
| Polymer degradation method | Highly hydrophilic polymer (such as PEG) interacts with water molecules around EV to form a hydrophobic environment, making EV precipitate | | Convenient operation | | The obtained EV has low purity and contains many polluting proteins, which has a great impact on the subsequent activity and function analysis | | (Savcı et al., 2021) | |
| Size-exclusion chromatography (SEC) | Using porous gel, EV was separated and purified through different molecular sizes. EV diameter is larger than protein, so EV is eluted first | | The purity is high and the structure is not easy to be damaged | | Time consuming, possibly mixed with protein or non-protein pollutants | | (Mehanny et al., 2022; Müller et al., 2021) | |
| Tangential flow filtration (TFF) | The solution enters the tangential flow ultrafiltration system and applies appropriate pressure to flow the solution from the high concentration side to the low concentration side. Ultrafiltration membranes only allow smaller molecules in solutes and solvents to pass through, while intercepting larger molecules and particles. | | Suitable for processing larger scale samples; Has good linear amplification; Can be classified and separated based on pore size | | Need to be used in conjunction with equipment, high cost; Reusable filters require regular cleaning and maintenance | | (Kim et al., 2020b) | |

###### Table S2. Necessary preparations and related research directions to ensure the safety of LBA-EVs.

| Item | Content |
| --- | --- |
| Strain selection and identification | Select lactic acid bacteria strains that are known to be safe and have probiotic properties, and conduct detailed identification, including genome sequencing and phenotype analysis. |
| In vitro safety assessment | Evaluate the cytotoxicity, immunogenicity, and potential pro-inflammatory properties of LAB-EVs in an in vitro model. |
| In vivo safety study | Conduct in vivo safety studies of LAB-EVs using animal models, including dose-response relationships, long-term safety of administration, and potential side effects. |
| Component analysis | Detailed analysis of proteins, lipids, nucleic acids, and other bioactive molecules in LAB-EVs to determine the safety of their components. |
| Production process control | Establish standardized LAB-EVs production and purification processes to ensure product consistency and traceability. |
| Stability study | Evaluate the stability of LAB-EVs under different storage conditions, including the effects of temperature and fermentation density. |
| Preclinical studies | Conduct preclinical studies in animal models to evaluate the health regulatory effects and safety of LAB-EVs. |
| Clinical trials | Conduct clinical trials to evaluate the safety, tolerability, and efficacy of LAB-EVs in the human body. |
| Risk assessment and management | Conduct a comprehensive risk assessment and develop corresponding risk management plans, including monitoring and handling of adverse events. |
| Regulatory compliance | Ensure that the development and application of LAB-EVs comply with relevant food safety and drug regulatory regulations. |
| Public education | Educate the public, raise awareness of the safety and potential health benefits of LAB-EVs, and maintain open communication channels. |
| Continuous monitoring | Even after the product is launched, its safety and effectiveness should be continuously monitored, and any potential issues should be identified and responded to in a timely manner. |

**References**

Cheng, K., Zhao, R., Li, Y., Qi, Y., Wang, Y., Zhang, Y., Qin, H., Qin, Y., Chen, L., Li, C., Liang, J., Li, Y., Xu, J., Han, X., Anderson, G. J., Shi, J., Ren, L., Zhao, X., & Nie, G. (2021). Bioengineered bacteria-derived outer membrane vesicles as a versatile antigen display platform for tumor vaccination via Plug-and-Display technology. *Nature Communications*, *12*(1), 2041. https://doi.org/10.1038/s41467-021-22308-8

Fan, J., Zhang, Y., Zuo, M., Ding, S., Li, J., Feng, S., Xiao, Y., & Tao, S. (2024). Novel mechanism by which extracellular vesicles derived from *Lactobacillus murinus* alleviates deoxynivalenol-induced intestinal barrier disruption. *Environment International*, *185*, 108525. https://doi.org/10.1016/j.envint.2024.108525

Harrison, N. A., Gardner, C. L., da Silva, D. R., Gonzalez, C. F., & Lorca, G. L. (2021). Identification of biomarkers for systemic distribution of nanovesicles from *Lactobacillus johnsonii* N6.2. *Frontiers in Immunology*, *12*, 723433. https://doi.org/10.3389/fimmu.2021.723433

Kim, W., Lee, E. J., Bae, I., Myoung, K., Kim, S. T., Park, P. J., Lee, K., Pham, A. V. Q., Ko, J., Oh, S. H., & Cho, E. (2020b). *Lactobacillus plantarum*-derived extracellular vesicles induce anti-inflammatory M2 macrophage polarization *in vitro*. *Journal of Extracellular Vesicles*, *9*(1), 1793514. https://doi.org/10.1080/20013078.2020.1793514

Mehanny, M., Kroniger, T., Koch, M., Hoppstädter, J., Becher, D., Kiemer, A. K., Lehr, C., & Fuhrmann, G. (2022). Yields and immunomodulatory effects of *Pneumococcal* membrane vesicles differ with the bacterial growth phase. *Advanced Healthcare Materials*, *11*(5), 2101151. https://doi.org/10.1002/adhm.202101151

Müller, L., Kuhn, T., Koch, M., & Fuhrmann, G. (2021). Stimulation of probiotic bacteria induces release of membrane vesicles with augmented anti-inflammatory activity. *ACS Applied Bio Materials*, *4*(5), 3739-3748. https://doi.org/10.1021/acsabm.0c01136

Rodovalho, V. de R., da Luz, B. S. R., Nicolas, A., do Carmo, F. L. R., Jardin, J., Briard-Bion, V., Jan, G., Le Loir, Y., de Carvalho Azevedo, V. A., & Guédon, E. (2021). Environmental conditions modulate the protein content and immunomodulatory activity of extracellular vesicles produced by the probiotic *Propionibacterium freudenreichii*. *Applied and Environmental Microbiology*, *87*(4), e02263-20. https://doi.org/10.1128/AEM.02263-20

Savcı, Y., Kırbaş, O. K., Bozkurt, B. T., Abdik, E. A., Taşlı, P. N., Şahin, F., & Abdik, H. (2021). Grapefruit-derived extracellular vesicles as a promising cell-free therapeutic tool for wound healing. *Food & Function*, *12*(11), 5144-5156. https://doi.org/10.1039/D0FO02953J

Tzipilevich, E., Habusha, M., & Ben-Yehuda, S. (2017). Acquisition of phage sensitivity by bacteria through exchange of phage receptors. *Cell*, *168*(1-2), 186-199. https://doi.org/10.1016/j.cell.2016.12.003
